# Asymmetric Fitness of Second-Generation Interspecific Hybrids Between *Ciona robusta* and *Ciona intestinalis*

**DOI:** 10.1534/g3.120.401427

**Published:** 2020-06-09

**Authors:** Naoyuki Ohta, Nicole Kaplan, James Tyler Ng, Basile Jules Gravez, Lionel Christiaen

**Affiliations:** Center for Developmental Genetics, Department of Biology, New York University, NY

**Keywords:** Tunicate, Ascidian, hybrids, reproductive isolation, speciation, evolution

## Abstract

Reproductive isolation is central to speciation, but interspecific crosses between two closely related species can produce viable and fertile hybrids. Two different species of tunicates in the same ascidian genus, *Ciona robusta* and *Ciona intestinalis*, can produce hybrids. However, wild sympatric populations display limited gene flow, suggesting the existence of obstacles to interspecific reproduction that remain unknown. Here, we took advantage of a closed culture system to cross *C. robusta* with *C. intestinalis* and established F1 and F2 hybrids. We monitored post-embryonic development, survival, and sexual maturation to characterize the genetic basis of simple traits, and further probe the physiological mechanisms underlying reproductive isolation. Partial viability of first and second generation hybrids suggested that both pre- and postzygotic mechanisms contributed to genomic incompatibilities in hybrids. We observed asymmetric fitness, whereby the *C. intestinalis* maternal lines fared more poorly in our system, pointing to maternal origins of species-specific sensitivity. We discuss the possibility that asymmetrical second generation inviability and infertility emerge from interspecific incompatibilities between the nuclear and mitochondrial genomes, or other maternal effect genes. This work paves the way to quantitative genetic approaches to study the mechanisms underlying genomic incompatibilities and other complex traits in the genome-enabled *Ciona* model.

Reproductive isolation is central to speciation (Mayr 1963; De Queiroz 2007), and results from the emergence of intrinsic or extrinsic barriers to reproduction that limit gene flow between populations (Seehausen *et al.* 2014). Prezygotic mechanisms of reproductive isolation, including habitat segregation, phenological and sexual isolation, hinder mating and/or fertilization between individuals of different species or populations undergoing speciation. Even when individuals overcome prezygotic obstacles to reproduction, postzygotic mechanisms can prevent growth and sexual maturation of hybrids. Intrinsic mechanisms of postzygotic reproductive isolation are referred to as genomic incompatibilities, also known as Bateson-Dobzhansky-Muller incompatibility (BDMI; (Orr 1996; Orr and Presgraves 2000; Presgraves 2010; Cutter 2012)). In addition, extrinsic postzygotic reproductive barriers, including interactions with the environment and with other individuals, can reduce the viability and/or fertility of the hybrid offspring. Despite obstacles to reproduction, individuals from closely related species occasionally produce viable and fertile hybrids, which in turn impact gene flow and species evolution, particularly through introgressions at specific loci between distinct genomes (Roux *et al.* 2013; Bouchemousse *et al.* 2016b).

Within the ascidian genus *Ciona*, distinct type A and type B were identified within the species *Ciona intestinalis*. These types were first thought to represent cryptic sub-species (Suzuki *et al.* 2005; Kano *et al.* 2006; Caputi *et al.* 2007; Nydam and Harrison 2010; Sato *et al.* 2012, 2014). However, they were more recently recognized as two distinct species, *Ciona robusta* and *Ciona intestinalis*, respectively (Brunetti *et al.* 2015). Based on molecular clock estimates, the speciation event that segregated *C. robusta* and *C. intestinalis* is thought to have occurred approximately 4 million years ago (Mya; (Nydam and Harrison 2007; Roux *et al.* 2013), following geographical separation between the Pacific and Atlantic oceans (Caputi *et al.* 2007; Bouchemousse *et al.* 2016b). However, the two species came in contact secondarily, and co-exist in the English Channel, where *C. intestinalis* is the endemic species, while *C. robusta* is thought to have invaded the area, in part through human transportation (Zhan *et al.* 2010; Nydam and Harrison 2011; Sato *et al.* 2012; Roux *et al.* 2013; Bouchemousse *et al.* 2016a). In the contact area, natural hybrids of *C. robusta* and *C. intestinalis* were found, but at very low frequencies. Furthermore, the two species displayed limited exchange of alleles (Nydam and Harrison 2011; Sato *et al.* 2012; Bouchemousse *et al.* 2016c), suggesting that mechanisms ensuring reproductive isolation largely restrict the expansion of hybrids, as well as gene flow between the two species in the contact region.

Mechanisms ensuring species-specific fertilization are important for prezygotic reproductive isolation (Mayr 1963; Seehausen *et al.* 2014; Herberg *et al.* 2018), but successful fertilization between *C. robusta* and *C. intestinalis* can routinely be obtained in the laboratory, despite indications that *C. intestinalis* sperm occasionally fails to fertilize *C. robusta* eggs (Suzuki *et al.* 2005; Sato *et al.* 2014; Bouchemousse *et al.* 2016a; Malfant *et al.* 2017). Notably, *Ciona* adults are self-incompatible hermaphrodites (Harada *et al.* 2008; Sawada *et al.* 2020), which spawn their gametes in the open water at dawn. Intrinsic prezygotic isolation would thus involve gamete recognition and/or fertilization success rather than, for example, mating behavior. Nonetheless, prezygotic reproductive isolation in *Ciona* may not suffice to explain the quasi-absence of natural hybrids and limited gene flow in the wild. Instead, it is thought that postzygotic mechanisms ensure reproductive isolation, including genomic incompatibility in the second generation hybrids. For instance, Sato and colleagues crossed F1 hybrids, produced by forcibly crossing *C. robusta* and *C. intestinalis*, and obtained backcrossed BC1 larvae (Sato *et al.* 2014). However, to our knowledge, the viability and fertility of F2 hybrids, which could provide clues about the physiological origins of the reproductive isolation between *Ciona robusta* and *Ciona intestinalis*, has not been reported.

In this study, we took advantage of a simple inland culture system to cross *C. robusta* and *C. intestinalis*, and maintain hybrids through multiple generations. We assayed survival, growth and sexual maturation, to further evaluate pre- and postzygotic mechanisms of reproductive isolation between *C. robusta* and *intestinalis*. Our observations indicate that F1 and F2 hybrids have reduced fitness compared to ​*C. robusta*, with a markedly reduced fertilization success and fitness in specific F2 crosses, suggesting the existence of mechanisms of reproductive isolation. ​Additionally, we report asymmetric second generation fitness, whereby both hybrids and homotypic animals born from ​*C. intestinalis*​ grandmothers fared poorly. This could be interpreted as inadequacy of our culture system for *C. intestinalis*​, but most likely reflect genomic incompatibilities between the nuclear ​*C. robusta*​ genome and maternally transmitted ​determinants from *C. intestinalis*, such as the mitochondrial genotype.

## Materials and Methods

### Animals

Wild-type *Ciona robusta* (*C. intestinalis* type A) and *Ciona intestinalis* (*C. intestinalis* type B) adults were collected in San Diego (CA) and Woods Hole (MA), respectively, and are within the range of known distribution for these species (Nydam and Harrison 2007; Caputi *et al.* 2007; Bouchemousse *et al.* 2016b). We confirmed species identification using established phenotypic criteria (Sato *et al.* 2012). Sperm and eggs were surgically obtained from mature animals, and used for controlled *in vitro* fertilizations to produce F1 generation animals, using established protocols (Christiaen *et al.* 2009). We cultured all animals at 18° (Sanyo, MIR-154), a presumed permissive temperature for both species, as well as for F1 hybrids (Sato *et al.* 2014; Malfant *et al.* 2017). Juveniles were kept in Petri dishes at 18° until 28 days post fertilization (dpf). We changed the buffered artificial sea water in the dishes and fed animals every other day. The young animals were transferred into a closed inland culture system at 28 dpf. We measured survival rate by counting the number of live animals in each Petri over time, and measured the size of each living individual from the tip of the siphon to the end of the body. The data were analyzed using Microsoft Office Excel and R. We dissected mature F1 animals to obtain sperm and/or eggs and generated F2 animals by controlled *in vitro* fertilization.

### Algae culture

We essentially followed an established protocol (Joly *et al.* 2007). We used two strains of microalgae, *Chaetoceros gracilis* and *Isochrysis galbana* (aka T.iso) as food for *Ciona* juveniles and adults, 10^7^ to 10^8^ cells for each Petri dish, and 10^9^ to 10^10^ cells for tanks. Stock, starter, and scale-up cultures of algae were kept in 250mL, 500mL and 2L flasks, respectively. Terminal food cultures were kept in 10L carboys. The flasks and carboys were maintained under constant light (Marineland), and were shaken once a day to prevent sedimentation. The cultures were inoculated every 10 to 14 days. Half of the cultures were diluted with autoclaved artificial sea water (Bio-Actif Salt, Tropic Marin) for the next round of cultures. We added 1mL Conway medium (Bouquet *et al.* 2009; Martí-Solans *et al.* 2015) and 1g sodium bicarbonate (Sigma Aldrich) per 1L artificial sea water, instead of bubbling CO_2_, to scale up intermediate and terminal food cultures. We added 1mL silicate solution (40g/L metasilicate sodium, Fisher Scientific) per 1L artificial sea water for scale-up and ongoing food cultures of *Chaetoceros*. Conway medium contains: 45g/L EDTA (C_10_H_16_N_2_O_8_, Acros Organics) 100g/L Sodium nitrate (NaNO_3_, Acros Organics), 33.3g/L Boric acid (H_3_BO_3_, Fisher Scientific), 20g/L Sodium dihydrogen phosphate (NaH_2_PO, Acros Organics), 1.5g/L Manganese (II) chloride (MnC_12_•4H_2_O, Acros Organics), 1.3g/L iron (III) chloride (FeCl_3_•6H_2_O, Acros Organics), 21mg/L Zinc chloride (ZnCl_2_, Acros Organics), 20mg/L Cobalt (II) chloride (CoCl_2_•6H_2_O, Acros Organics), 10mg/L Ammonium heptamolybdate ((NH_4_)_6_Mo_7_O_24_•4H_2_O, Acros Organics), 21mg/L Copper (II) sulfate (CuSO_4_•5H_2_O, Acros Organics), 10mg/L Thiamine (Acros Organics), 10mg/L Cyanocobalamin (Acros Organics), 200μg/L Biotin (Fisher Scientific).

### System maintenance

The culture system held 20L glass aquarium tanks (Carolina), 5L polypropylene beakers (Midland Scientific) and 2L polycarbonate aquarium tanks (Eisco), which each could hold 16, 4 and 2 Petri dishes, respectively (Supplemental Figure S1A-C). The 20L tanks, 5L beakers and 2L small tanks were set in an 18° Chamber. Sea water (Bio-Actif Salt, Tropic Marin) was controlled by bio-balls (Biomate, Lifegard Aquatics) seeded with bacteria (BioDigest, Prodibio), and salinity was set at 34 ppt. The 20L tanks were cleaned twice a week, and the 5L beakers and the 2L small tanks were cleaned three times a week. The 20L tanks, 5L beakers and 2L small tanks each efficiently supported animal growth and sexual maturation (Supplemental Figure S1D-E).

### Genotyping

F1 juveniles were taken from Petri dishes and digested by proteinase (Thermo Fisher Science) to obtain genomic DNA as described previously (Ohta *et al.* 2010). Oral siphon and sperm were surgically obtained from mature animals, and processed for genomic DNA extraction using the QIAamp DNA Micro Kit (Qiagen), or digested with proteinase. The genomic DNA was used for PCR amplification (35 cycles of 95° 30’’, 58° 30’’, 72° 1’) of target regions with Ex Taq HS DNA polymerase (Takara Bio). The PCR products were purified enzymatically with ExoSAP-IT Express PCR product cleanup (Thermo Fisher Scientific), or by NucleoSpin Gel and PCR clean-up (Macherey-Nagel). PCR products were sequenced by Genewiz. The primers used in this study are summarized in Supplemental Table S1.

### Data availability

The authors affirm that all data necessary for confirming the conclusions of the article are present within the article, figures, and tables. Supplemental material available at figshare: https://doi.org/10.25387/g3.12014826.

## Results

### Reciprocal crosses between Ciona robusta and C. intestinalis produce hybrids

**Figure 1:**
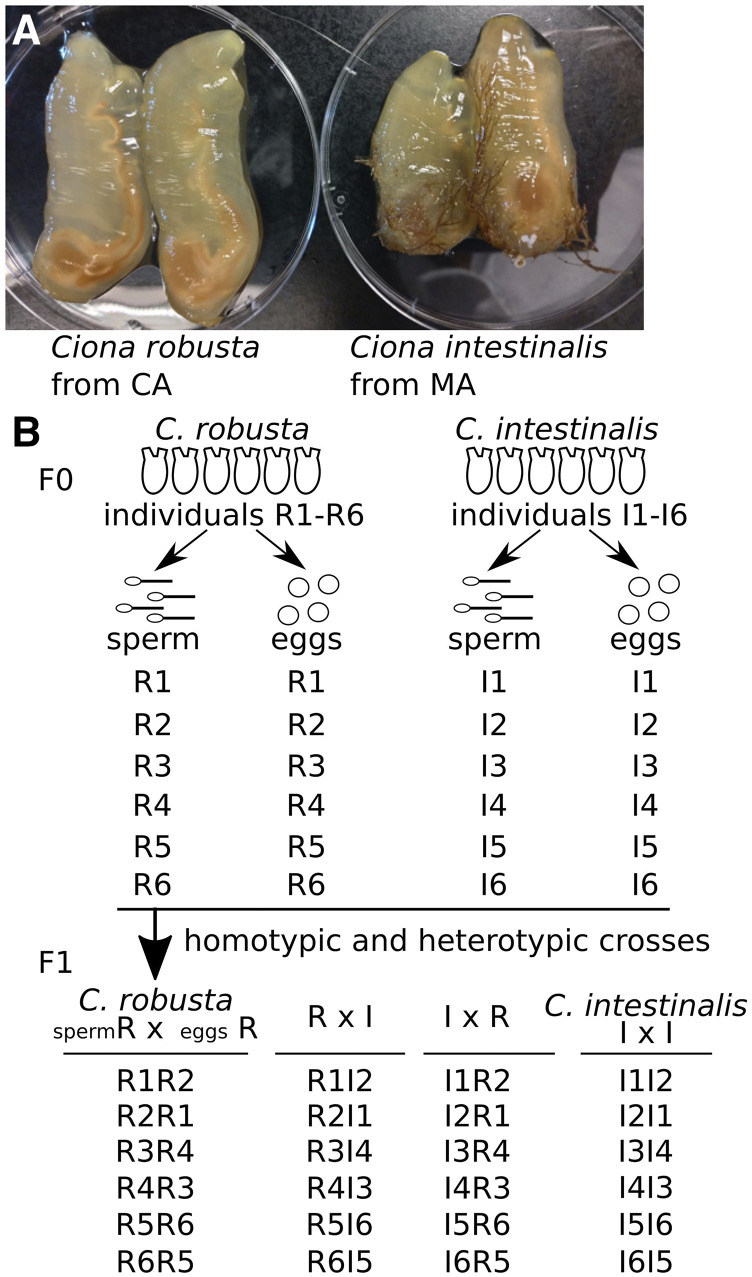
Crossing animals to make genetic hybrids. (A) Mature animals of *Ciona robusta* and *Ciona intestinalis* were collected in San Diego (CA) and Woods Hole (MA), respectively. The animals were in 100 mm Petri dishes. (B) Six animals were dissected to obtain sperm R1 to R6 and eggs R1 to R6 of *C. robusta* and sperm I1 to I6 and eggs I1 to I6 of *C. intestinalis*. These sperm and the eggs were homo- and heterotypic crossed to make *C. robusta* (sperm R x eggs R), RxI hybrid, IxR hybrid and *C. intestinalis* (IxI) types.

**Figure 2:**
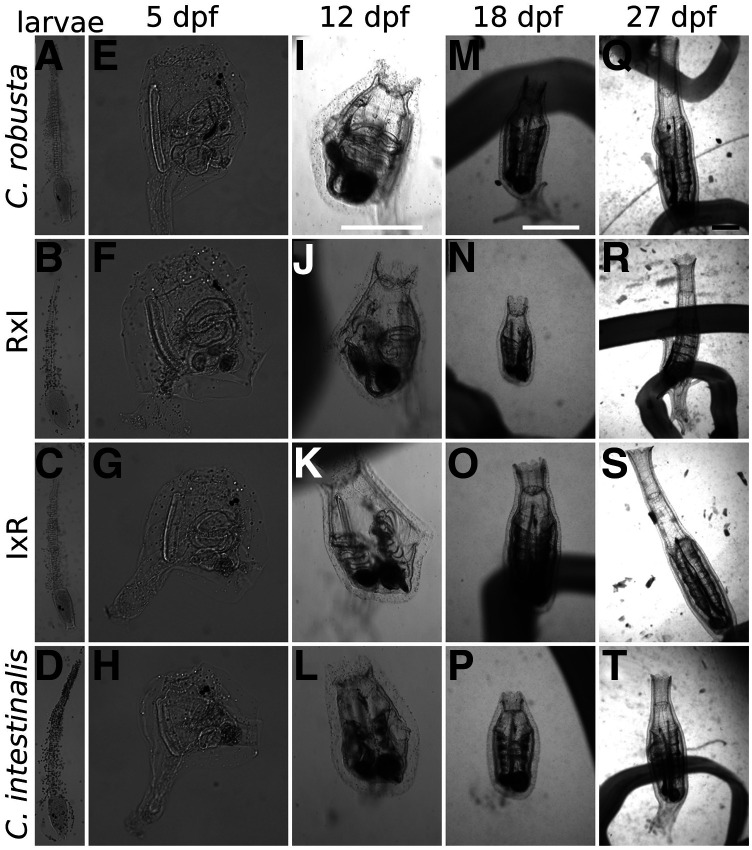
Development of F1 animals. (A-H) Images taken after fixation in formamide of: swimming larvae (A-D) and 5 dpf juveniles (E-H). (I-T) Images of living animals were taken under a microscope of: 12 dpf juveniles (I-L), 18 dpf young adults (M-P) and 27 dpf young adults (Q-T). Scale bars in I, M and Q show 1 mm.

In order to cross *Ciona robusta* and *Ciona intestinalis*, we obtained mature animals from San Diego (CA) and Woods Hole (MA), respectively (Figure 1A). Using six isolated batches of sperm and eggs from each species, we performed homotypic and heterotypic crosses by *in vitro* fertilization to obtain twenty-four combinations of four types of animals in three separate partial diallels: the parental strains *C. robusta* and *C. intestinalis*, and reciprocal F1 hybrids, which we termed RxI, and IxR, for hybrids obtained from *C. robusta* sperm and *C. intestinalis* eggs, or *C. intestinalis* sperm and *C. robusta* eggs, respectively (Figure 1B). We obtained hundreds of swimming larvae from each cross (Figure 2A-D), and did not estimate fertilization rates or the proportion of hatched larvae, although this contrasts with previous studies, which suggested that *C. robusta* oocytes were largely refractory to fertilization by *C. intestinalis* sperm (Suzuki *et al.* 2005; Bouchemousse *et al.* 2016a; Malfant *et al.* 2018). Further work will be required to determine whether these discrepancies stem from biological and/or experimental differences between studies.

**Figure 3:**
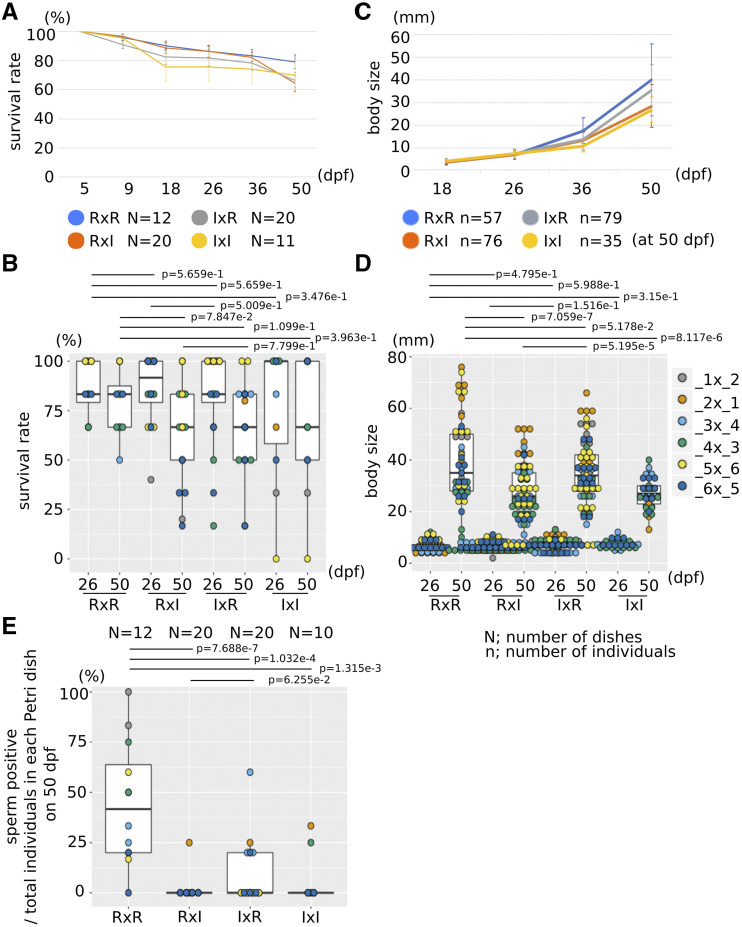
Growth of F1 animals. (A) The chart shows the average survival rate in each Petri dish. Error bars show standard deviation. (B) The dot and boxplot shows the survival rate in each Petri dish at 26 and 50 dpf. Color shows each Petri dish in each parental combination. (C) The chart shows the average size of each individual. Error bars show standard deviation. (D) The dot and boxplot shows the size of each individual at 26 and 50 dpf. Color shows each individual in each parental combination. (E) The dot and boxplot shows the ratio of animals which had sperm at 50 dpf in each Petri dish. N shows the numbers of Petri dishes. n shows the numbers of individuals at 50 dpf. p values were calculated by an ANOVA.

We monitored development following hatching, settlement, metamorphosis and initial growth, and did not observe obvious differences between the four types, although this cursory analysis may have missed subtle quantitative variability (Figure 2E-T). We measured survival rates from 5 to 50 days post fertilization (dpf) by counting the number of animals in each Petri dish (Figure 3A-B). About 70% of animals in all four conditions survived to 50 dpf (Figure 3A-B), and an ANOVA did not show significant differences in survival rate between the four types at 26 and 50 dpf, except for between *C. robusta* and RxI hybrid at 50 dpf (Figure 3B). Notably, there were no significant differences in the survival rate between F1 RxI and IxR hybrids at 26 or 50 dpf. We monitored the size of animals from 18 dpf to 50 dpf, while keeping the feeding regime constant across conditions (Figure 3C-D). Here too, an ANOVA did not reveal significant differences in the size of F1 hybrids at 26 dpf, although size significantly differed between hybrids and *C. robusta* at 50 dpf (Figure 3D). Notably, an ANOVA did not show significant size differences between F1 RxI and IxR hybrids at 26 dpf, but showed it at 50dpf. A previous study reported differences in growth rate for hybrid animals of 28 dpf (Malfant *et al.* 2018). Taken together, these observations suggest that reciprocal first generation hybrids of *C. robusta* and *C. intestinalis* are generally as healthy as the parental strains, as they did not display marked differences in post-hatching survival and growth. However, we observed subtle but significant differences between animals obtained from *C. robusta* or *C. intestinalis* eggs, whether through homotypic or heterotypic crosses. This observation echoes a previous report that *C. robusta* eggs confer higher tolerance to challenging water temperatures (Sato *et al.* 2015), and is a harbinger of asymmetries observed in subsequent crosses (see below).

Next, we sought to raise *Ciona* hybrids to sexual maturity in our experimental facility. In a previous report, Sato and colleagues cultured F1 hybrids in their natural environment, the English Channel, where the two species live in sympatry, and obtained mature animals after a couple of months (Sato *et al.* 2014). Here, we took advantage of a custom inland culture system to raise and monitor animals through sexual maturation. By 50 dpf, half of the *C. robusta* individuals were producing sperm, whereas that proportion dropped significantly for the other groups of animals (Figure 3E). The observation that *C. intestinalis* F1 animals also fail to produce gametes points to a defect in fitness possibly arising from a specific inadequacy with our culture system, and prevents us from strictly interpreting the lower fitness of RxI and IxR hybrids in terms of genomic incompatibility. We kept these animals until they produced eggs and/or sperm, which we collected surgically, thus sacrificing F1 animals, to test their fertility and obtain F2 animals (Table 1).

**Table 1:** Summary of F1 animals

	*C. robusta*	RxI	IxR	*C. intestinalis*
sperm+	14	16	21	7
used for F2	6	13	18	6
discarded	8	3	3	1
egg+	2	6	13	0
used for F2	0	6	11	0
discarded	2	0	2	0
dead or unknown	7	39	33	0
discarded	36	20	22	28
total	57	76	79	35

### Phenotypes of hybrid adult animals

**Figure 4:**
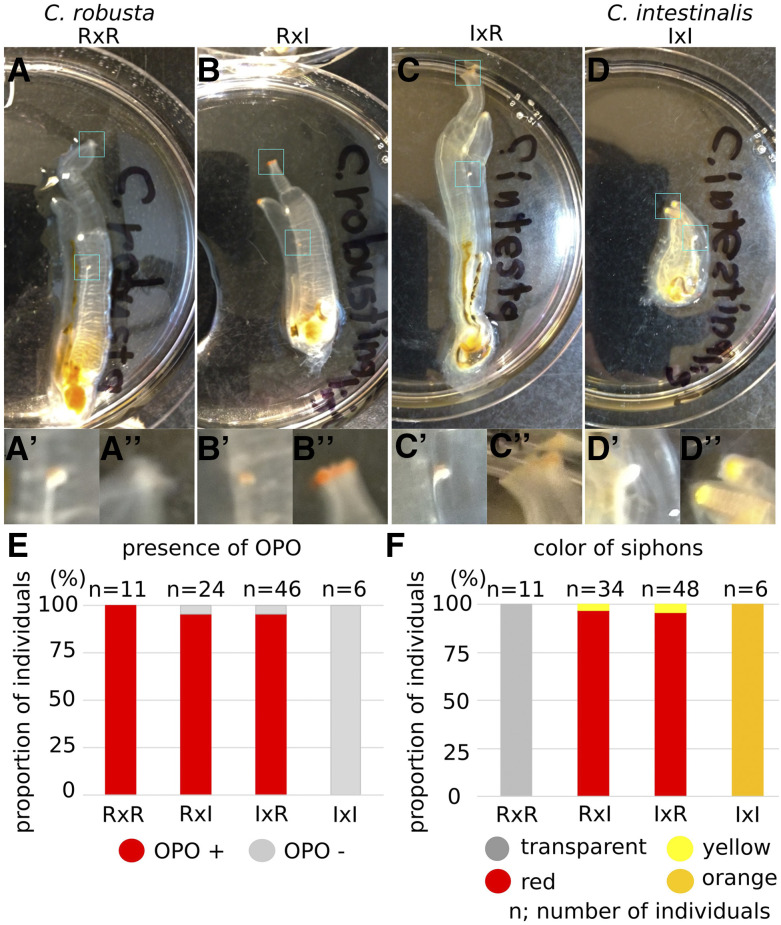
Phenotype of F1 mature animals. (A) Images were taken of F1 mature animals in 100 mm Petri dishes; *C. robusta* (A), RxI hybrid (B), IxR hybrid (C) and *C. intestinalis* (D). (A’-D’, A’’-D’’) The tip of sperm duct (A’-D’) and oral siphon (A’’-D’’) of each individual are shown in the insets. (E) The bar shows the proportion of individuals having OPO at the tip of sperm duct in F1 mature animals. (F) The bar shows the proportion of individuals having color in the rim of oral and atrial siphons in F1 mature animals. n shows the numbers of individuals.

One obvious difference between parental species is the presence of an orange pigment organ (OPO) at the tip of the sperm duct in *C. robusta*, but not in *C. intestinali*s (Millar 1953; Hoshino and Tokioka 1967; Ohta *et al.* 2010; Sato *et al.* 2012, 2014; Tajima *et al.* 2019). F1 animals from our parental strains did recapitulate this species-specific trait (Figure 4A, A’, D and D’). For both RxI and IxR hybrids, the majority of animals had OPO at the tip of the sperm duct (Figure. 4B, B’, C, C’ and E), in agreement with a previous report (Sato *et al.* 2014), thus indicating that OPO formation is a dominant trait.

Another character that differs between *Ciona* species is the color of siphons (Sato *et al.* 2012), whereby *C. intestinalis* has yellow and orange pigmentation around the tip of siphons that is lacking in *C. robusta* (Figure. 4A, A’’, D and D’’), although this feature was deemed quite variable and taxonomically unreliable (Brunetti *et al.* 2015). As for the OPO, the majority of RxI and IxR hybrids displayed a bright red pigmentation at the rim of oral and atrial siphons (Figure 4B, B’’, C, C’’ and F), also consistent with a previous report (Sato *et al.* 2014). The observation that siphon pigmentation displays an overdominant phenotype in hybrids is consistent with its lack of reliability for taxonomic purposes. Further work will be required to determine how proposed species-specific and taxonomically informative traits, such as tubercular prominences in the siphons (Brunetti *et al.* 2015), which we could not observe clearly, are inherited through generations of hybrids.

### Genotyping of hybrid animals

**Figure 5:**
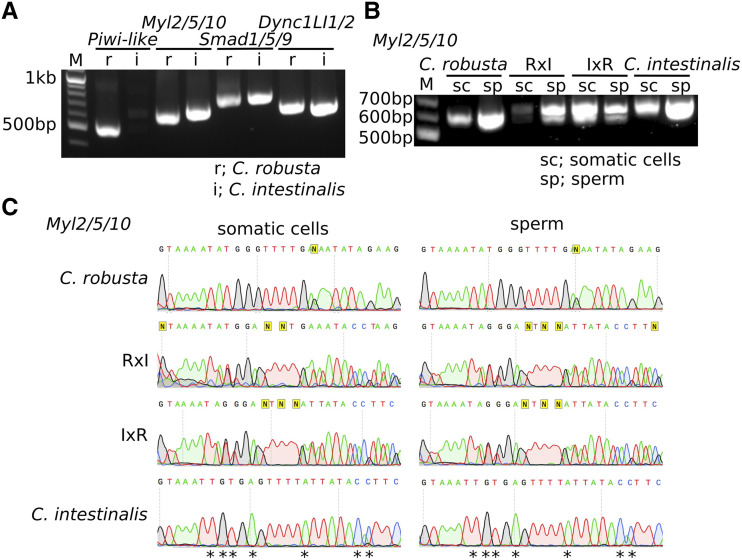
Genotype of F1 animals. (A) PCR was done with primers designed at *Piwi-like*, *Myl2/5/10*, *Smad1/5/9* and *Dync1LI1/2* gene loci from genomic DNAs from sperm of F0 mature animals of *C. robusta* and *C. intestinalis*. (B) PCR was done with primers designed at *Myl2/5/10* gene locus. Genomic DNA was collected from somatic tissue in oral siphon and sperm of F1 mature adults; *C. robusta*, RxI hybrid, IxR hybrid and *intestinalis*. (C) The sequence of PCR products in (B) were read by *Myl2/5/10*-sequence primer. The asterisks show parts of peaks distinct between *C. robusta* and C. *intestinalis*.

The distribution of variable traits in homo- and heterospecific crosses suggested that RxI and IxR F1 animals are *bona fide* hybrids. As a complement to phenotypic characterization, and to rule out cross-contaminations during the *in vitro* fertilization procedure, we sought to perform molecular genetics analyses to assay the distribution of species-specific marker alleles in the different families (Suzuki *et al.* 2005; Nydam and Harrison 2007). We unsuccessfully tested two primer sets, markers 1 and 2, which were previously used to distinguish *C. robusta* and *C. intestinalis* ((Suzuki *et al.* 2005); Supplemental Figure S2A-C). However, sequence differences between the PCR products distinguished between species-specific alleles (Supplemental Figure S2D). As an alternative, we used a primer set designed at *Myosin light chain 2/5/10* (*Myl2/5/10*; KH.C8.239) locus, which could distinguish *C. robusta* and *C. intestinalis* alleles by the size difference of PCR products (Figure 5A, Supplemental Figure S3A-B). Sequencing amplicons showed conserved 6^th^ and 7^th^ exons, but an indel in the 6^th^ intron that distinguished alleles from different species (Supplemental Figure S3C). We isolated genomic DNA from three F1 juvenile individuals from each type. Six juveniles of either *C. robusta* or *C. intestinalis* yielded single bands, albeit of higher molecular weight for the latter (Supplemental Figure S3D). By contrast, six juveniles of either RxI or IxR crosses yielded double bands, showing that these animals had both *C. robusta* and *C. intestinalis Myl2/5/10* alleles, and were indeed hybrids. Consistent with electrophoresis patterns, sequence analysis revealed single alleles for either *C. robusta* or *C. intestinalis*, whereas F1 RxI and IxR hybrids produced a mixture of *C. robusta*- and *C. intestinalis*-specific sequences (Supplemental Figure S3E). Of note, genomic DNA from both somatic tissue and gametes yielded similar results, whereby homotypic *C. robusta* and *C. intestinalis* produced single PCR bands in different sizes, while those of both hybrids produced double PCR bands (Figure 5B). Taken together with the results of phenotypic observations, genotyping data indicated that F1 RxI and IxR animals were *bona fide* hybrids.

### Backcrossing to Ciona robusta eggs

**Figure 6:**
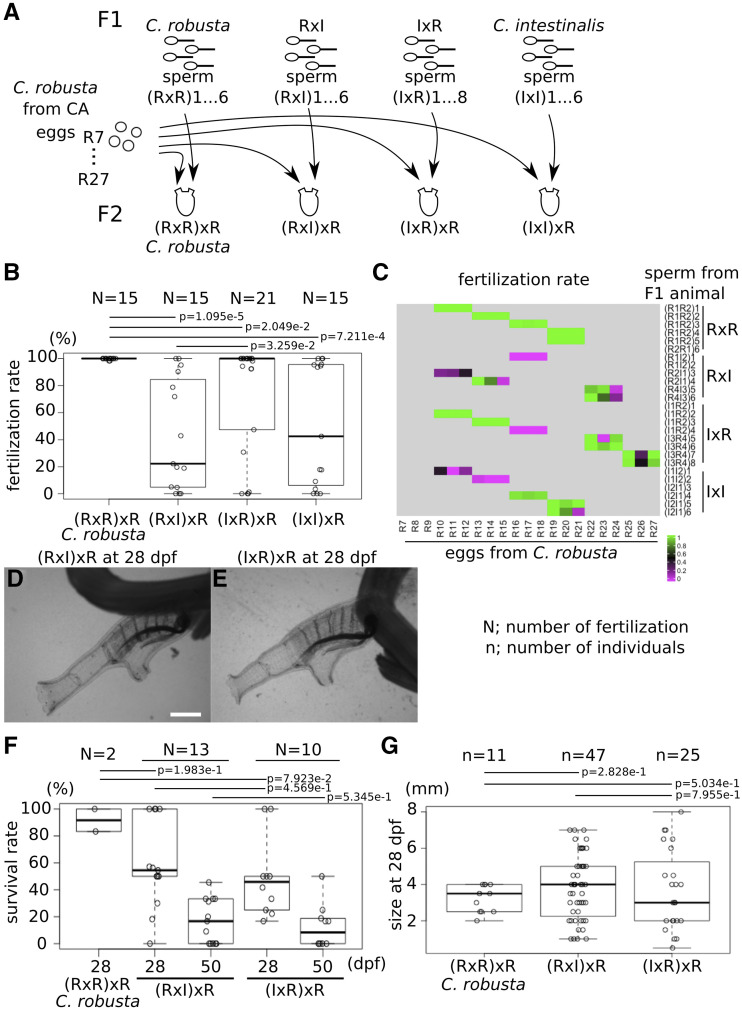
Backcrossing to *C. robusta* eggs. (A) Sperm of (RxR)1 to 6, (RxI)1 to 6, (IxR)1 to 8 and (IxI)1 to 6 were collected from F1 *C. robusta*, RxI hybrid, IxR hybrid and *C. intestinalis* mature animals, respectively. Wildtype eggs R7 to R27 were collected from mature animals in CA. (B) The dot and boxplot shows the fertilization rate. (C) The heatmap shows the fertilization rate in each fertilization. (D, E) Young adults of F2 (RxI)xR (D) and (IxR)xR (E) at 28 dpf were imaged under a microscope. Scale bar shows 1 mm. (F) The dot and boxplot shows the survival rate of each fertilization. (G) The dot and boxplot shows the size of each individual at 28 dpf. N shows the numbers of fertilization. n shows the numbers of individuals. p values were calculated by an ANOVA.

Since we could grow F1 hybrids to sexual maturity, we sought to test whether their sperm, which appeared first, could fertilize wildtype *C. robusta* eggs. For this backcross experiment, we collected sperm from 6, 6, 8 and 6 mature F1 animals of *C. robusta*, RxI and IxR hybrids, and *C. intestinalis*, respectively (Figure 6A, Tables 1-2). On the other hand, we obtained wildtype eggs from 21 (R7-27) mature *C. robusta* animals. We crossed these sperm and eggs in 78 different combinations (summarized in Table 2). Because F2 (IxI)xR hybrids were potentially equivalent to F1 IxR hybrids, we did not analyze them further. We raised F2 *C. robusta* animals by crossing sperm from F1 *C. robusta* (RxR) animals and eggs from *C. robusta* collected from the wild, and kept F2 *C. robusta* animals as controls. We counted the proportion of fertilized eggs out of total eggs to score fertilization rates (Figure 6B-C). The fertilization rates for *C. robusta* were almost 100%, while the rates dropped and varied between 0–100% in the other crosses (Figure 6C). Notably, the sperm of F1 RxI hybrid appeared less potent to fertilize *C. robusta* eggs than that of F1 IxR hybrids, which is reminiscent of previously reported difficulties in using *C. robusta* eggs in interspecific fertilizations. A heatmap of the fertilization rates showed that there were no infertile eggs from wildtype *C. robusta*, while sperm from (R1I2)1, (R2I1)3, (I1R2)4, (I1I2)1 and (I1I2)2 might have been sterile (Figure 6C). While the fertilization rates depend on the quantity and/or quality of sperm, which we did not measure, the range of observed fertilization rates suggested variable compatibilities between specific combinations of sperm and eggs. These observations indicated that F1 hybrids of *C. robusta* and *C. intestinalis* can produce fertile sperm capable of fertilizing *C. robusta* eggs with variable efficacy, which likely constitutes a first, prezygotic, obstacle to interspecific reproduction and gene flow.

**Table 2:** Summary of BC1 animals

F1 type	Father	Mother	Fertilization rate	Hatched larvae	Survival rate (28dpf)	(28dpf/5dpf)	Survival rate (50dpf)	(50dpf/5dpf)	Size at 28dpf (mm)
*C. robusta*	(R1R2)1	R10	100.0%	1000<	N.A		N.A		
	(R1R2)1	R11	100.0%	1000<	N.A		N.A		
	(R1R2)1	R12	100.0%	1000<	N.A		N.A		
	(R1R2)2	R13	100.0%	1000<	100%	(6/6)	N.A		2.5,2,2.5,3,2.5,4
	(R1R2)2	R14	100.0%	1000<	83.3%	(5/6)	N.A		3.5,4,3.5,4,4
	(R1R2)2	R15	100.0%	1000<	N.A		N.A		
	(R1R2)3	R16	100.0%	1000<	N.A		N.A		
	(R1R2)3	R17	98.2%	1000<	N.A		N.A		
	(R1R2)3	R18	100.0%	1000<	N.A		N.A		
	(R1R2)4	R19	100.0%	1000<	N.A		N.A		
	(R1R2)4	R20	100.0%	1000<	N.A		N.A		
	(R1R2)4	R21	100.0%	1000<	N.A		N.A		
	(R1R2)5	R19	100.0%	1000<	N.A		N.A		
	(R1R2)5	R20	100.0%	1000<	N.A		N.A		
	(R1R2)5	R21	100.0%	1000<	N.A		N.A		
	(R2R1)6	R7	N.A	1000<	N.A		N.A		
	(R2R1)6	R8	N.A	1000<	N.A		N.A		
	(R2R1)6	R9	N.A	1000<	N.A		N.A		
RxI	(R1I2)1	R16	0.0%	200	N.A		N.A		
	(R1I2)1	R17	0.0%	100	N.A		N.A		
	(R1I2)1	R18	0.0%	100	N.A		N.A		
	(R2I1)2	R7	N.A	14	0%	(0/4)	0%	(0/4)	N.A
	(R2I1)2	R8	N.A	257	56.3%	(9/16)	31.3%	(5/16)	4,7,5,2,1,3,6,2,4
	(R2I1)2	R9	N.A	36	50.0%	(1/2)	0%	(0/2)	2
	(R2I1)2	R10	19.6%	500<	54.5%	(6/11)	45.5%	(5/11)	3,4,1.5,7,5,4
	(R2I1)3	R11	22.2%	500<	30.0%	(3/10)	20.0%	(2/10)	2,6,6
	(R2I1)3	R12	42.9%	500<	18.2%	(2/11)	9.1%	(1/11)	4.5,3.5
	(R2I1)4	R13	100.0%	300<	100%	(3/3)	33.3%	(1/3)	1.5,7,1
	(R2I1)4	R14	78.7%	300<	57.1%	(4/7)	0%	(0/7)	1.5,2,2,2.5
	(R2I1)4	R15	4.7%	200	50.0%	(2/4)	0%	(0/4)	5,3.5
	(R4I3)5	R22	90.3%	1000<	50.0%	(3/6)	16.7%	(1/6)	5,2.5,3.5
	(R4I3)5	R23	95.2%	1000<	100%	(2/2)	0%	(0/2)	1,3
	(R4I3)5	R24	5.0%	<100	N.A		N.A		
	(R4I3)6	R22	100.0%	1000<	100%	(6/6)	33.3%	(2/6)	4,3,5,3,6,6.5
	(R4I3)6	R23	71.8%	1000<	100%	(6/6)	33.3%	(2/6)	6.5,4,6,4.5,3,4
	(R4I3)6	R24	18.9%	200	N.A		N.A		
IxR	(I1R2)1	R7	N.A	0	N.A		N.A		
	(I1R2)1	R8	N.A	0	N.A		N.A		
	(I1R2)1	R9	N.A	3	N.A		N.A		
	(I1R2)2	R10	100.0%	1000<	41.7%	(5/12)	16.7%	(2/12)	4.5,1,2,1.5,3
	(I1R2)2	R11	100.0%	1000<	25.0%	(4/16)	18.8%	(3/16)	2,4,8,2
	(I1R2)2	R12	100.0%	1000<	16.7%	(1/6)	16.7%	(1/6)	1.5
	(I1R2)3	R13	100.0%	500<	50.0%	(2/4)	25.0%	(1/4)	2,6.5
	(I1R2)3	R14	100.0%	1000<	22.2%	(2/9)	0%	(0/9)	3,4
	(I1R2)3	R15	100.0%	1000<	100%	(2/2)	0%	(0/2)	3,6
	(I1R2)4	R16	0.0%	2	N.A		N.A		
	(I1R2)4	R17	0.0%	1	N.A		N.A		
	(I1R2)4	R18	0.0%	4	N.A		N.A		
	(I3R4)5	R22	100.0%	1000<	100%	(4/4)	0%	(0/4)	6.5,7,2,7
	(I3R4)5	R23	1.3%	100	N.A		N.A		
	(I3R4)5	R24	94.3%	1000<	N.A		N.A		
	(I3R4)6	R22	100.0%	1000<	33.3%	(1/3)	0%	(0/3)	0.5
	(I3R4)6	R23	92.4%	1000<	N.A		N.A		
	(I3R4)6	R24	100.0%	1000<	N.A		N.A		
	(I3R4)7	R25	100.0%	1000<	50.0%	(1/2)	0%	(0/2)	1
	(I3R4)7	R26	30.8%	500<	N.A		N.A		
	(I3R4)7	R27	100.0%	1000<	N.A		N.A		
	(I3R4)8	R25	98.2%	1000<	50.0%	(3/6)	50.0%	(3/6)	4,4,5
	(I3R4)8	R26	47.4%	500<	N.A		N.A		
	(I3R4)8	R27	92.3%	1000<	N.A		N.A		
*C. intestinalis*	(I1I2)1	R10	42.4%	1000<	N.A		N.A		
	(I1I2)1	R11	0.0%	20	N.A		N.A		
	(I1I2)1	R12	17.6%	200	N.A		N.A		
	(I1I2)2	R13	3.4%	70	N.A		N.A		
	(I1I2)2	R14	0.0%	2	N.A		N.A		
	(I1I2)2	R15	0.0%	1	N.A		N.A		
	(I2I1)3	R7	N.A	18	N.A		N.A		
	(I2I1)3	R8	N.A	26	N.A		N.A		
	(I2I1)3	R9	N.A	9	N.A		N.A		
	(I2I1)4	R16	95.8%	1000<	N.A		N.A		
	(I2I1)4	R17	93.5%	1000<	N.A		N.A		
	(I2I1)4	R18	95.3%	1000<	N.A		N.A		
	(I2I1)5	R19	100.0%	1000<	N.A		N.A		
	(I2I1)5	R20	94.5%	1000<	N.A		N.A		
	(I2I1)5	R21	100.0%	1000<	N.A		N.A		
	(I2I1)6	R19	100.0%	1000<	N.A		N.A		
	(I2I1)6	R20	82.1%	1000<	N.A		N.A		
	(I2I1)6	R21	9.1%	10	N.A		N.A		

Sato and colleagues also successfully obtained mature F1 hybrids, which could be backcrossed to parental species, and the backcrossed BC1 hybrids could develop into seemingly normal larvae (Sato *et al.* 2014). Likewise, we raised BC1 (RxI)xR and (IxR)xR hybrids at 18°, and allowed them to metamorphose and become young adults by 28 dpf (Figure 6D-E). As a measure of hybrid fitness, we calculated survival rates by counting the number of animals that survived to 28 and 50 dpf relative to the numbers of juveniles at 5 dpf (Figure 6F). Only half of BC1 (RxI)xR and (IxR)xR hybrid juveniles survived to 28 dpf, compared to almost 90% for *C. robusta*. Approximately 20% of juveniles of both BC1 hybrids survived to 50 dpf. Both BC1 (RxI)xR and (IxR)xR hybrids had lower survival rates than F2 *C. robusta* animals, while an ANOVA did not show significant differences in survival rate on 28 and 50 dpf between (RxI)xR and (IxR)xR hybrids. These observations suggest that BC1 hybrid juveniles experience higher mortality rates, consistent with proposed genomic incompatibilities in second generation hybrids (Dobzhansky-Müller Incompatibilities, DMI, (Malfant *et al.* 2018)). However, in the absence of homotypic *C. intestinalis* controls, we cannot formally exclude the possibility that the presence of *C. intestinalis* alleles altered the fitness of hybrid animals in our culturing system, independently of incompatibilities with the *C. robusta* genome.

As a complement to survival, we measured the body size of BC1 animals at 28 dpf (Figure 6G). The size of F2 *C. robusta* juveniles varied between 2 and 4 mm (average = 3.23, SD = 0.75, n = 11), while the size of BC1 (RxI)xR and (IxR)xR hybrids varied between 0.5 and 8 mm ((RxI)xR; average = 3.82, SD = 1.78, n = 47, (IxR)xR; average = 3.70, SD = 2.22, n = 25). This suggested that growth rates are more variable in the BC1 hybrid population, as expected following the segregation of alleles for a likely multigenic trait such as individual growth rate.

**Figure 7:**
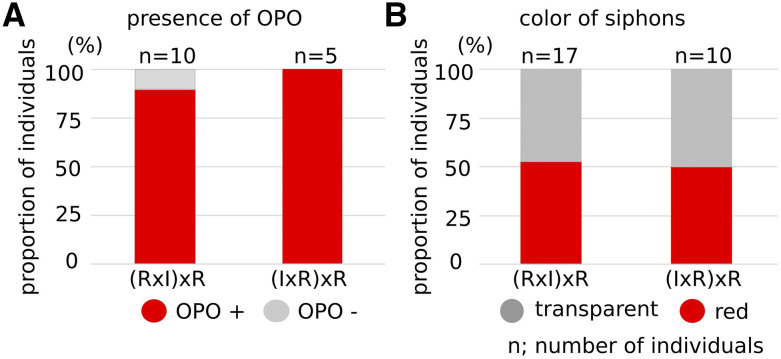
Phenotype of BC1 hybrid mature animals. (A) The bar shows the proportion of individuals having OPO at the tip of sperm duct in BC1 hybrid mature animals. (B) The bar shows the proportion of individuals having color in the rim of oral and atrial siphons in BC1 hybrid mature animals. n shows the numbers of animals.

Seventeen and ten individuals of (RxI)xR and (IxR)xR hybrids grew to mature adults, respectively, thus allowing us to observe the presence of OPO and the color of their siphons (Figure 7 and Table 3). Except for one individual [(R2I1)2xR8], BC1 (RxI)xR and (IxR)xR hybrids had OPO at the tip of the sperm duct (Figure 7A and Table 3), which is also consistent with the presence of OPO being a dominant *C. robusta* trait. Half of the individuals in both BC1 (RxI)xR and (IxR)xR hybrids had red color in the rim of siphons, as did F1 hybrids, while the other half had transparent siphons, the same as normal *C. robusta* (Figure 7B). This could be explained considering a single gene, with distinct *C. robusta* and *C. intestinalis* alleles, which coexist in F1 hybrids and segregate with a 1:1 ratio in the BC1 hybrid population, because animals heterozygous for *C. robusta* and *C. intestinalis* alleles should produce red-colored siphons, as seen in F1 hybrids, while homozygous *C. robusta* alleles should produce colorless siphons.

**Table 3:** Phenotype of BC1 animals

Type	BC1 individuals	Siphon	OPO	Gamete
(RxI)xR	(R2I1)2xR8	Transparent	N.A	
	(R2I1)2xR8	Red	+	
	(R2I1)2xR8	Red	N.A	
	(R2I1)2xR8	Transparent	+	Sperm+
	(R2I1)2xR8	Transparent	—	Sperm+, eggs+
	(R2I1)2xR10	Transparent	+	Sperm+
	(R2I1)2xR10	Transparent	+	
	(R2I1)2xR10	Transparent	+	
	(R2I1)3xR11	Transparent	+	
	(R2I1)3xR11	Transparent	+	
	(R2I1)3xR12	Red	N.A	
	(R2I1)4xR13	Red	+	Sperm+
	(R4I3)5xR22	Red	N.A	
	(R4I3)6xR22	Red	N.A	
	(R4I3)6xR22	Red	+	
	(R4I3)6xR23	Red	N.A	
	(R4I3)6xR23	Red	N.A	
(IxR)xR	(I1R2)2xR10	Transparent	+	
	(I1R2)2xR11	Red	+	Sperm+
	(I1R2)2xR11	Red	+	
	(I1R2)3xR13	Transparent	+	
	(I3R4)5xR22	Red	N.A	
	(I3R4)5xR22	Red	N.A	
	(I3R4)5xR22	Transparent	N.A	
	(I3R4)8xR25	Transparent	+	
	(I3R4)8xR25	Transparent	N.A	
	(I3R4)8xR25	Red	N.A	

Finally, both (RxI)xR and (IxR)xR BC1 hybrids grew and matured to produce sperm and eggs (Table 3 and Supplemental Table S2). The sperm could fertilize *C. robusta* eggs to produce BC2 hybrids, which survived at least 28 dpf, after which we stopped observations. This indicates that the BC1 hybrids that survive, grow and mature are potentially fertile. This possibility is not incompatible with the existence of DMI. Instead, it is consistent with the existence of defined hotspots of unidirectional introgression observed in wild populations (Roux *et al.* 2013).

### Inbreeding F1 RxI and IxR hybrids

**Figure 8:**
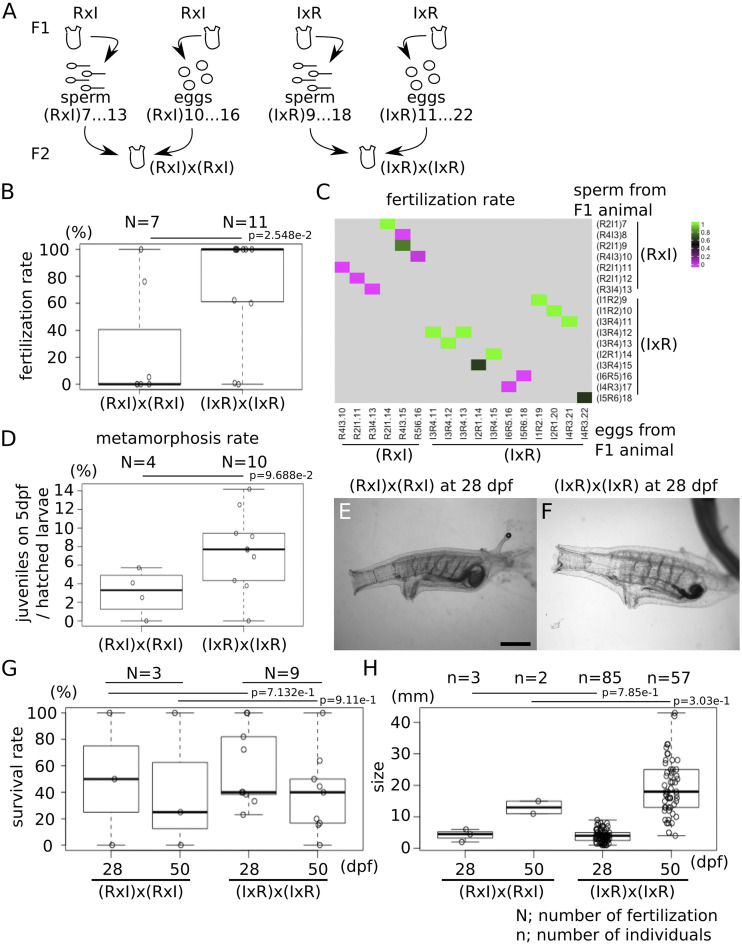
Inbreeding of F1 RxI and IxR hybrids. (A) Sperm and eggs were collected from F1 RxI hybrids of (RxI)7 to 13 and (RxI)10 to 16, respectively. Sperm and eggs were collected from F1 IxR hybrids of (IxR)9 to 18 and (IxR)11 to 22, respectively. These sperm and eggs were crossed to produce F2 hybrids. (B) The dot and boxplot shows the fertilization rate. (C) The heatmap shows the fertilization rate in each fertilization. (D) The dot and boxplot shows the metamorphosis rates. (E, F) Young adults of F2 RxI (E) and IxR (F) hybrids at 28 dpf were imaged under a microscope. Scale bar = 1 mm. (G) The dot and boxplot shows the survival rate of each fertilization. (H) The dot and boxplot shows the size of individuals at 28 and 50 dpf. N shows the numbers of fertilization. n shows the number of individuals. p values were calculated by an ANOVA.

Next, we leveraged the fertility of *C. robusta* x *C. intestinalis* offspring to test whether crossing F1 hybrids would yield viable F2 animals, which would in principle provide opportunities for quantitative genetics approaches for the analysis of complex traits. We obtained sperm from 7 and 10 individuals, and eggs from 7 and 11 F1 RxI and IxR mature animals, respectively, and used them for within-type fertilizations (Figure 8A, Tables 1 and 4). Fertilization rates were significantly higher for IxR hybrids than for RxI hybrids (Figure 8B). Specifically, crosses between IxR hybrids yielded almost 100% fertilization in 11 trials, except for two combinations, (I6R5)16x(I5R6)18 and (I4R3)17x(I6R5)16, while crosses between RxI hybrids almost invariably failed, except for the (R2I1)7x(R2I1)14 combination (Figure 8C). The data suggested that the (I6R5)16 F1 adult produced unhealthy gametes, because neither sperm nor eggs yielded productive fertilization. By contrast with backcrossing fertilizations, a limited number of eggs from F1 hybrids produced only hundreds of hatched larvae, thus limiting the numbers of F2 hybrid juveniles in each Petri dish (Table 4). Thus, we calculated metamorphosis rates of F2 hybrids by counting the number of juveniles relative to the number of swimming larvae for each fertilization, and could thus evaluate 4 and 10 fertilizations for RxI and IxR crosses, respectively (Figure 8D). The metamorphosis rates of F2 RxI and IxR hybrids ranged from 0 to 6% and 14%, respectively, which were lower than for *C. robusta* in regular fertilization (2–80%, average = 25.6%, SD = 15.6, N = 33). Notably, an ANOVA showed significant differences in the metamorphosis rate between F2 RxI and IxR hybrids. Because of low fertilization and metamorphosis rates, we obtained only 7 F2 RxI hybrid juveniles by 5 dpf, compared to 137 F2 IxR hybrid juveniles (Table 4). In total, 3 and 85 juveniles of F2 RxI and IxR hybrids survived to 28 dpf, and displayed normal morphologies, similar to *C. robusta* (Figure 8E-F). There were no obvious morphological differences among 28 dpf F2 hybrid individuals between cross types. Survival rates were calculated by counting the number of individuals that survived to 28 dpf and 50 dpf, relative to the number of juveniles at 5 dpf (Figure 8G). Only 1 juvenile from the (R2I1)9x(R4I3)15 and (R4I3)10x(R5I6)16 crosses survived to 50 dpf, and there were only 2 individuals of F2 RxI hybrids that survived to 50 dpf, compared to 57 F2 IxR hybrid individuals, indicating that F2 hybrids were less viable in the RxI type than in the IxR type. This intriguing observation suggested the existence of asymmetric second generation genomic incompatibilities. However, in the absence of F2 homotypic *C. intestinalis* controls, we cannot exclude the possibility that the presence of *C. intestinalis* alleles in the RxI line reduces the fitness of F2 animals in our system, regardless of incompatibilities between genotypes. In either case, the observed asymmetry could involve maternal determinants such as the mitochondrial genome, assuming quasi-exclusive maternal inheritance of the mitochondrial DNA (Nishikata *et al.* 1987), whereby the *C.intestinalis* mitochondrial lineage in RxI families lowers their fitness in our culturing system (see discussion).

**Table 4:** Summary of F2 animals

F1 hybrids	Father	Mother	Fertilization rate	Hatched larvae	Survival rate (28dpf)	(28dpf/5dpf)	Survival rate (50dpf)	(50dpf/5dpf)	size at 28dpf (mm)	size at 50dpf (mm)
RxI	(R2I1)7	(R2I1)14	100%	49 larvae	0%	(0/2)	0%	(0/2)	N.A	N.A
	(R4I3)8	(R4I3)15	0%	1 larva	N.A		N.A			
	(R2I1)9	(R4I3)15	76%	40 larvae	100%	(1/1)	100%	(1/1)	4.5	11
	(R4I3)10	(R5I6)16	5.30%	70 larvae	50.0%	(2/4)	25.0%	(1/4)	6,2	15
	(R2I1)11	(R4I3)10	0%	0 larvae	N.A		N.A			
	(R2I1)12	(R2I1)11	0%	0 larvae	N.A		N.A			
	(R3I4)13	(R3I4)13	0%	0 larvae	N.A		N.A			
IxR	(I1R2)9	(I1R2)19	100%	About 300 larvae	23.1%	(3/13)	0%	(0/13)	1.5,1.5,1.5	N.A
	(I1R2)10	(I2R1)20	100%	335 larvae	38.5%	(10/26)	15.4%	(4/26)	2,3.5,5,9,4,3,2,3,3.5,2	8,12,20,43
	(I3R4)11	(I4R3)21	100%	About 800 larvae	82.0%	(50/61)	63.9%	(39/61)	3,7,6,3.5,4,4,2.5,4,4,4,2,1.5,5,8,4,4, 8,6,4,4,3,5,6,4,3,8,2,1,1.5,4,4,4,7,3, 1,6,7,3,7.5,5,1,5,6,1.5,2.5,3,3.5,2,5,3	10,23,27,4,18,15,20,28,14,32,16,18,16, 21,42,33,23,23,9,8,28,30,24,18,13,30, 15,33,21,25,19,25,16,21,8,22,17,9,12
	(I3R4)12	(I3R4)11	100%	48 larvae	33.3%	(2/6)	16.7%	(1/6)	3,2.5	13
	(I3R4)12	(I3R4)13	100%	58 larvae	40.0%	(2/5)	40.0%	(2/5)	5,6	12,12
	(I3R4)13	(I3R4)12	100%	127 larvae	72.2%	(13/18)	44.4%	(8/18)	6,1,1,4,4,4,2,3.5,2,4,4,7,2	16,16,17,5,18,13,13,25
	(I2R1)14	(I3R4)15	100%	11 larvae	100%	(1/1)	100%	(1/1)	4	24
	(I3R4)15	(I2R1)14	62.20%	29 larvae	100%	(2/2)	50.0%	(1/2)	3,3	17
	(I6R5)16	(I5R6)18	0%	0 larvae	N.A		N.A			
	(I4R3)17	(I6R5)16	0.84%	3 larvae	N.A		N.A			
	(I5R6)18	(I4R3)22	60%	53 larvae	40.0%	(2/5)	20.0%	(1/5)	5,5	11

We could measure body sizes for only 3 and 2 F2 RxI hybrid individuals at 28 and 50 dpf, preventing robust statistical analysis. By contrast, 85 and 57 F2 IxR hybrid individuals measured at 28 and 50 dpf showed a range of body sizes similar to that of BC1 hybrids (Figure 8H). This is also consistent with the notion that body size is a polygenic trait, which displays increased continuous phenotypic variation following alleles segregation of multiple genes in F2. This also suggests that these IxR animals are not obviously subjected to second generation genomic incompatibilities. Finally, although body size is likely multifactorial, *i.e.*, influenced by the environment, especially the availability of food, we surmise that most of the observed variation in controlled laboratory conditions is due to polygenic effects.

**Figure 9:**
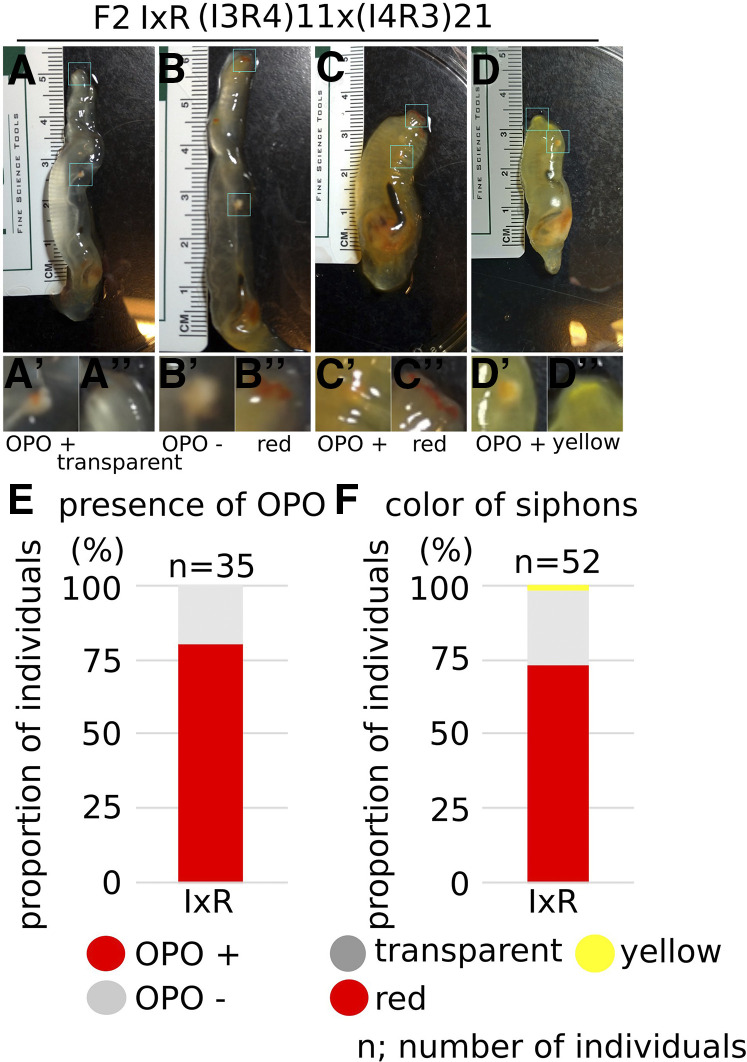
Phenotype of F2 mature animals. (A-D) Images were taken of four individuals of (I3R4)11x(I4R3)21 F2 IxR mature animals. (A’-D’, A’’-D’’) The tip of sperm duct (A’-D’) and oral siphon (A’’-D’’) of each individual are shown in the insets. (E) The bar shows the proportion of individuals having OPO at the tip of sperm duct in F2 IxR mature animals. (F) The bar shows the proportion of individuals having color in the rim of oral and atrial siphons in F2 IxR mature animals. n shows the numbers of individuals.

One and fifty-three individuals of F2 RxI and IxR hybrids grew to become mature adults, respectively, thus allowing us to observe the presence of OPO and the color of their siphons (Figure 9 and Table 5). The F2 RxI hybrid individual, (R4I3)10x(R5I6), had red color in the rim of siphons. Three-quarters of the individuals in F2 IxR hybrids had OPO at the tip of the sperm duct (Figure 9E and Table 5), which is also consistent with the presence of OPO being a dominant *C. robusta* trait. Three-quarters of the F2 IxR hybrids had red color in the rim of siphons, as did F1 hybrids, while the other quarter had transparent siphons, the same as normal *C. robusta* (Figure 9F). These proportions are also consistent with mendelian segregation of monogenic traits with dominant species-specific alleles.

**Table 5:** Phenotype of F2 animals

Type	F2 type	dpf	Size (mm)	OS	OPO	Sperm	
RI	(R4I3)10x(R5I6)16	60	26	Red	N.A	—	
IR	(I3R4)11x(I4R3)21	50	10	Transparent	N.A	—	
	(I3R4)11x(I4R3)21	50	4	Transparent	N.A	—	
	(I3R4)11x(I4R3)21	50	9	Red	N.A	—	
	(I3R4)11x(I4R3)21	50	8	Transparent	N.A	—	
	(I3R4)11x(I4R3)21	50	8	Red	N.A	—	
	(I3R4)11x(I4R3)21	50	9	Red	N.A	—	
	(I3R4)12x(I3R4)13	50	12	Red	N.A	—	
	(I3R4)13x(I3R4)12	50	5	N.A	N.A	—	
	(I5R6)18x(I4R3)22	54	10	Red	+	+	
	(I3R4)12x(I3R4)11	63	11	Transparent	+	—	
	(I3R4)11x(I4R3)21	65	12	Red	N.A	—	
	(I3R4)11x(I4R3)21	65	20	Red	+	—	
	(I3R4)11x(I4R3)21	65	18	Red	+	+	
	(I3R4)12x(I3R4)13	65	15	Red	N.A	—	
	(I3R4)13x(I3R4)12	65	12	Red	N.A	—	
	(I3R4)13x(I3R4)12	65	16	Transparent	+	—	
	(I3R4)13x(I3R4)12	65	11	Red	+	—	
	(I2R1)14x(I3R4)15	66	27	Red	+	+	
	(I3R4)15x(I2R1)14	66	13	Red	N.A	—	Egg+
	(I3R4)11x(I4R3)21	70	18	Red	+	+	
	(I3R4)11x(I4R3)21	70	23	Transparent	+	+	
	(I3R4)11x(I4R3)21	70	35	Transparent	N.A	—	
	(I3R4)11x(I4R3)21	70	26	Red	N.A	—	
	(I3R4)11x(I4R3)21	70	47	Red	N.A	—	
	(I3R4)13x(I3R4)12	70	14	Red	+	—	Egg+
	(I3R4)13x(I3R4)12	70	13	Red	N.A	—	
	(I3R4)11x(I4R3)21	70	42	Red	+	+	
	(I3R4)11x(I4R3)21	70	30	Red	+	+	
	(I3R4)11x(I4R3)21	70	27	Transparent	—	+	Sperm_IR23
	(I3R4)11x(I4R3)21	70	30	Red	+	—	
	(I3R4)11x(I4R3)21	70	35	Red	—	+	Egg+; egg_IR28
	(I3R4)13x(I3R4)12	70	10	Red	+	—	
	(I1R2)10x(I2R1)20	73	24	Red	N.A	—	
	(I3R4)13x(I3R4)12	75	45	Red	+	+	
	(I3R4)11x(I4R3)21	75	38	Red	—	+	
	(I3R4)11x(I4R3)21	75	53	Transparent	+	+	Egg+; egg_IR29, yellow body
	(I3R4)11x(I4R3)21	75	32	Red	+	+	Sperm_IR24
	(I3R4)11x(I4R3)21	75	44	Red	N.A	—	
	(I3R4)11x(I4R3)21	75	45	Red	—	+	Egg+; egg_IR30
	(I3R4)11x(I4R3)21	75	60	Red	—	+	Sperm_IR25
	(I3R4)11x(I4R3)21	75	23	Red	—	+	
	(I3R4)11x(I4R3)21	75	33	Yellow	+	+	Yellow body
	(I3R4)11x(I4R3)21	75	16	Transparent	+	—	
	(I3R4)11x(I4R3)21	75	50	Transparent	+	+	
	(I3R4)11x(I4R3)21	75	33	Red	+	+	Yellow body
	(I3R4)11x(I4R3)21	75	33	Transparent	+	+	
	(I3R4)11x(I4R3)21	79	38	Red	—	+	Egg+, yellow body
	(I3R4)11x(I4R3)21	79	70	Red	+	+	Sperm_IR26
	(I3R4)11x(I4R3)21	79	50	Red	+	+	Egg+; egg_IR32
	(I3R4)11x(I4R3)21	79	53	Transparent	+	+	Sperm_IR27
	(I3R4)11x(I4R3)21	79	80	Red	+	+	
	(I3R4)11x(I4R3)21	79	54	Red	+	+	Egg+; egg_IR31
	(I1R2)10x(I2R1)20	82	65	Red	+	+	

Finally, F2 IxR hybrids grew and matured to produce sperm and eggs (Table 5 and Supplemental table S3). The sperm and eggs could fertilize each other to produce F3 IxR hybrids, which survived at least 28 dpf, after which we stopped observations. This indicates that at least the F2 IxR hybrids that survive, grow and mature are fertile, opening future possibilities for inland cultures of hybrid lines.

**Figure 10:**
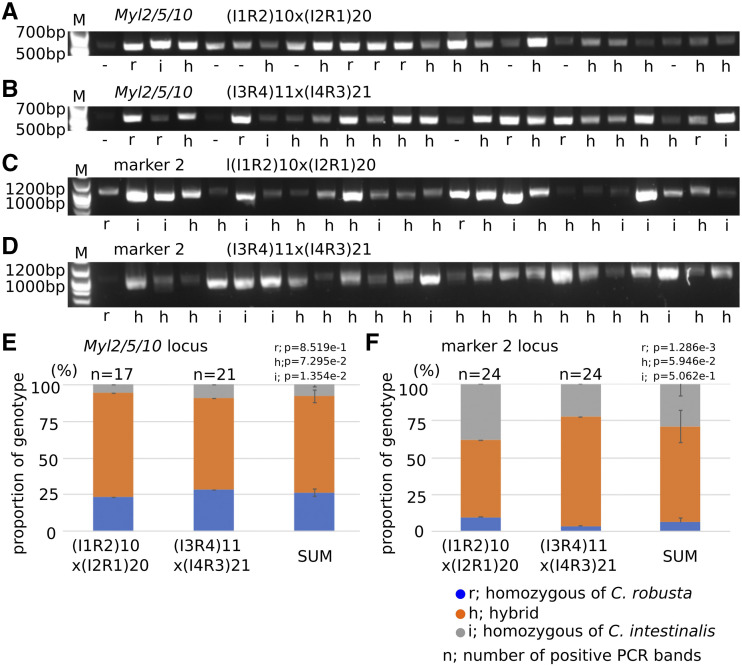
Genotyping of F2 IxR hybrid. (A-D) PCR was done with primers designed at *Myl2/5/10* (A, B) and marker 2 (Suzuki *et al.* 2005) gene loci. Genomic DNAs were collected from F2 larvae; each 24 larva from (I1R2)10x(I2R1)20 (A, C) and (I3R4)11x(I4R3)21 (B, D). (E, F) The bar shows the proportion of genotypes at *Myl2/5/10* (E) and marker 2 (F) gene loci in (I1R2)10x(I2R1)20 and (I3R4)11x(I4R3)21, and sum of two. p values were calculated by z-test from theoretical predicted value, 25% or 50%.

Following Mendel’s laws, the proportions of homo- and heterozygous animals among F2 hybrids should follow a 1:2:1 distribution in the absence of hybrid dysgenesis, inbreeding depression and/or second generation incompatibilities. We analyzed the genotypes at *Myl2/5/10* and marker 2 loci for 24 swimming larvae in each of two lines of F2 IxR hybrids, ((I1R2)10x(I2R1)20 and (I3R4)11x(I4R3)21) (Figure 10). All the PCR amplicons were verified by sequencing, which informed formal genotyping. At the *Myl2/5/10* locus, there were 4 and 6 larvae showing homozygous *C. robusta* alleles, 12 and 13 heterozygous larvae, and 1 and 2 larvae homozygous for the *C. intestinalis* allele out of 17 and 21 verified samples in (I1R2)10x(I2R1)20 and (I3R4)11x(I4R3)21, respectively (Figure 10E). The proportion of *C. intestinalis* genotype was significantly different from the theoretical estimation 25% (*P* = 1.354e-2 by z-test). By contrast, at the marker 2 locus, there were 2 and 1 larvae homozygous for the *C. robusta* allele, 13 and 18 heterozygous larvae, and 9 and 5 larvae homozygous for the *C. intestinalis* allele out of 24 and 24 verified samples in (I1R2)10x(I2R1)20 and (I3R4)11x(I4R3)21, respectively (Figure 10F). At this locus, the proportion of *C. robusta* genotype was significantly different from the theoretical estimation 25% (*P* = 1.286e-3 by z-test). Biased genotype in *Myl2/5/10* showing less *C. intestinalis* type and *marker 2* showing less *C. robusta type*, suggests that these genes of homozygous type are linked to loci depleted in F2 hybrids populations. Because *Myl2/5/10* and *marker 2* genes are on different chromosomes and neither are located in the inferred hotspots of introgression (Roux *et al.* 2013), their allelic distributions might be independent and differentially affected by linkage with incompatible loci. Future work will be required to characterize the genetic underpinnings of genomic incompatibilities between *Ciona* species, their sensitivity to environmental conditions (including culturing systems), their relationships to documented “hotspots” of introgression (Roux *et al.* 2013), and their impact on speciation.

## Discussion

In this study, we crossed the ascidian species *Ciona robusta* and *Ciona intestinalis* to establish hybrid lines, further probe the reproductive isolation of these recently distinguished species, and explore opportunities for quantitative genetics using the genome-enabled *Ciona* model. Taking advantage of a simple inland culture system, we monitored post-embryonic development and survival, and successfully raised F1 and F2 hybrid and backcrossed animals to maturity. The partial viability of first and second generation hybrids provided insights into the genetics of simple traits, such as the presence of OPO, which appears to be a dominant *C. robusta*-specific trait. On the other hand, siphon pigmentation showed an overdominant phenotype in hybrids, suggesting more complex genetic interactions, although the trait distribution in F2 hybrids could be explained by allele segregation at one locus. Moreover, simple quantitative traits, such as body size, showed an increased variability in F2 hybrids as expected for polygenic traits following allele segregation. These observations suggest that quantitative genetics approaches could be used to study complex traits that differ between *C. intestinalis* and *C. robusta*, such as tolerance to high water temperature (Caputi *et al.* 2015; Malfant *et al.* 2017).

Despite preliminary evidence of allele segregation in F2 hybrids, the representation of genotype combinations is likely to be biased due to genomic incompatibilities, which might hinder quantitative analysis of polygenic traits. Indeed, our observations suggest that both pre- and postzygotic mechanisms contribute to genomic incompatibilities in hybrids, and thus act as obstacles to interspecific reproduction between these two *Ciona* species. These observations are consistent with previous reports (Nydam and Harrison 2011; Sato *et al.* 2012; Bouchemousse *et al.* 2016a; c). Nonetheless, the incomplete penetrance of first and second generation incompatibilities suggests that certain combinations of *C. robusta* and *C. intestinalis* genotypes are viable, which would permit at least low levels of gene flow between populations, and is consistent with the existence of previously reported hotspots of introgression (Roux *et al.* 2013).

Asymmetric fertilization success in reciprocal interspecific crosses (Turelli and Moyle 2007) was previously observed between *Ciona robusta* and *Ciona intestinalis* (Suzuki *et al.* 2005; Bouchemousse *et al.* 2016a; Malfant *et al.* 2018), but the mechanisms remain elusive. On the other hand, asymmetric second generation inviability and infertility points to the mitochondrial genome as the most likely source of reduced fitness in *C. intestinalis* maternal lineages. Indeed, this asymmetry suggested that mechanisms of genomic incompatibility involve interactions between the nuclear genomes and maternal determinants inherited in a *trans*-generational manner, such as mitochondrial DNA (Turelli and Moyle 2007; Burton and Barreto 2012; Sloan *et al.* 2017). However, in the absence of homotypic F2 *C. intestinalis* animals, we cannot formally rule out the possibility that the *C. intestinalis* maternal lineage itself causes reduced fitness in our culture system, independently of alleged incompatibilities between the *C. robusta* and the *C. intestinalis* genomes. Thoroughly addressing this possibility will require the development of culturing conditions more compatible with *C. intestinalis*.

Nonetheless, if low *C. intestinalis* fitness sufficed to explain our results, we would expect to also observe a markedly lower fitness of F1 RxI hybrids compared to F1 IxR hybrids, which emerge from the *C. robusta* maternal lineage. To the contrary, we could obtain F1 RxI hybrids and raise them to maturity to produce backcrossed animals, which suggested a more pronounced reduction of fitness in second generation RxI hybrids from the *C. intestinalis* maternal lineage. Moreover, we reasoned that the second generation in the RxI lineage, which necessary follows gametogenesis and possible recombinations between *C. robusta* and *C. intestinalis* chromosomes in F1 RxI hybrids, is the first generation where the *C. intestinalis* mitochondrial genome could encounter homozygous *C. robusta* alleles in the nuclear genome, which encodes the majority of mitochondrial proteins. For instance, incompatibilities between the nuclear and mitochondrial genomes in hybrids were reported in various organisms, including fungi (Lee *et al.* 2008; Presgraves 2010), insect (Meiklejohn *et al.* 2013; Hoekstra *et al.* 2013), nematode (Chang *et al.* 2016) and mammals (Ma *et al.* 2016). For these reasons, and despite the lack of F2 IxI controls, we favor the hypothesis that asymmetric second generation incompatibilities between the *C. robusta* and *C. intestinalis* genomes limit the fitness of the RxI hybrid lineage, in a way that depends on the environment, on possible maternal effects genes and/or on specific interactions between the nuclear and mitochondrial genomes. Finally, it is tempting to speculate that these asymmetric incompatibilities provide a mechanistic explanation for the unidirectional introgressions observed in wild populations (Roux *et al.* 2013).
